# PTP1B negatively regulates nitric oxide-mediated *Pseudomonas aeruginosa* killing by neutrophils

**DOI:** 10.1371/journal.pone.0222753

**Published:** 2019-09-18

**Authors:** Lei Yue, Min Yan, Michel L. Tremblay, Tong-Jun Lin, Hua Li, Ting Yang, Xia Song, Tianhong Xie, Zhongping Xie

**Affiliations:** 1 The Institute of Medical Biology, Chinese Academy of Medical Sciences and Peking Union Medical College, Kunming, Yunnan, China; 2 Department of Microbiology and Immunology, Kunming Medical University, Kunming, Yunnan, China; 3 Rosalind and Morris Goodman Cancer Research Centre, Department of Biochemistry, McGill University, Montréal, Quebec, Canada; 4 Department of Microbiology and Immunology, Dalhousie University, Halifax, Nova Scotia, Canada; 5 Department of Pediatrics, Dalhousie University, Halifax, Nova Scotia, Canada; Louisiana State University, UNITED STATES

## Abstract

Neutrophils play a critical role in host defense against *Pseudomonas aeruginosa* infection. Mechanisms underlying the negative regulation of neutrophil function in bacterial clearance remain incompletely defined. Here, we demonstrate that protein tyrosine phosphatase-1B (PTP1B) is a negative regulator of *P*. *aeruginosa* clearance by neutrophils. PTP1B-deficient neutrophils display greatly enhanced bacterial phagocytosis and killing, which are accompanied by increased Toll-like receptor 4 (TLR4) signaling activation and nitric oxide (NO) production following *P*. *aeruginosa* infection. Interestingly, PTP1B deficiency mainly upregulates the production of IL-6 and IFN-β, leads to enhanced TLR4-dependent STAT1 activation and iNOS expression by neutrophils following *P*. *aeruginosa* infection. Further studies reveal that PTP1B and STAT1 are physically associated. These findings demonstrate a negative regulatory mechanism in neutrophil underlying the elimination of *P*. *aeruginosa* infection though a PTP1B-STAT1 interaction.

## Introduction

*Pseudomonas aeruginosa* is a prevalent opportunistic pathogen that is the common cause of exacerbations of chronic obstructive pulmonary disease (COPD)[1] and community acquired pneumonia (CAP)[2]. It is also the predominant pathogen-based cause of morbidity and mortality in cystic fibrosis (CF) patients[3, 4]. The innate immune response plays a critical role in host defense against *P*. *aeruginosa* infection[5]. This immune process requires the effective production of cytokines and chemokines to recruit neutrophils to inflammatory sites, which culminates in the phagocytosis and killing of the bacterium[6, 7]. A key factor for controlling *P*. *aeruginosa* is the maintenance of a balanced immune response, which effectively eliminates *P*. *aeruginosa* without causing detrimental inflammation and tissue damage[8, 9]. However, the mechanisms remain incompletely defined.

Neutrophils are an important line of host defense to combat *P*. *aeruginosa* pulmonary infection[10]. Neutrophils express all tested TLRs except TLR3, which are activated by bacterial pathogen-associated molecular patterns (PAMPs) and can induce downstream signaling pathways[11] that lead to the formation of phagosomes and lysosomes that kill bacteria. The oxidative attack on phagocytosed microbes, that occurs in neutrophils, employs highly toxic reactive oxygen species (ROS) and reactive nitrogen species (RNS), which damage intracellular components and kill extracellular pathogens[12]. *P*. *aeruginosa* has also evolved strategies to impair the bactericidal function of ROS[13]. Nitric oxide (NO), as the major effector of RNS, can eliminate bacteria, especially *P*. *aeruginosa* resistant to ROS[14].

NO production relies on transcriptional activation of the inducible nitric oxide synthase (iNOS) gene. The expression of iNOS is activated by pathogens binding to TLRs and requires the participation of multiple downstream cytokines and transcription factors[15, 16]. The synthesis of NO in neutrophils is regulated by tightly controlled epigenetic modifications, in which phosphorylation and dephosphorylation are fundamental mechanisms of expression regulation[17]. The coordinated actions of protein tyrosine kinases and protein tyrosine phosphatases determine the level of tyrosine phosphorylation in a reversible manner[18].

PTP1B belongs to the protein tyrosine phosphatase family, and its activity is sensitive to a wide variety of extracellular stimuli, such as insulin, growth factor signaling and amino acid starvation[19]. Roles for PTP1B in inflammation and innate immunity have also been demonstrated. Xu et al. reported a negative regulatory role for PTP1B in response to various TLR ligands which through inhibition of MyD88, TRIF, IRF3 and STAT1 dependent pathways[20]. Regulatory role for PTP1B has been proposed in the STATs signaling pathway. PTP1B has been shown to dephosphorylated the JAK2 and Tyk2[21], as well as STAT3[22], exerting a negative effect on activation of the pathway.

We have demonstrated the pivotal role of protein tyrosine phosphatase-1B (PTP1B) in resisting *P*. *aeruginosa* lung infection [23]. Our findings showed that in PTP1B-deficient mice, the clearance of *P*. *aeruginosa* was significantly enhanced due to neutrophil recruitment. However, whether PTP1B is important in direct killing of *P*. *aeruginosa* by neutrophils have not been reported previously.

In this study, we employed an *in vitro* neutrophil model to demonstrate that PTP1B negatively regulates the phagocytosis and NO-dependent killing of *P*. *aeruginosa*. This process is mediated by the TLR4-STAT1-iNOS signaling pathway. Importantly, we revealed that STAT1 is the target of PTP1B regulation. Hence, our study illustrates the key activities of PTP1B in neutrophil resistance to *P*. *aeruginosa* infection.

## Materials and methods

### Animals

PTP1B-deficient mice (C57BL/6 background) were originally provided by Michel L. Tremblay (McGill Cancer Centre, Montréal, QC, Canada). Animal care and experimental protocols were reviewed and approved by the Yunnan Provincial Experimental Animal Management Association and the Experimental Animal Ethics Committee of the Institute of Medical Biology, Chinese Academy of Medical Sciences, according to the national guidelines on animal work in China. The animals were housed in specific pathogen free facilities and anesthetized with ketamine to minimize pain during relevant procedures.

### Antibodies

Antibodies against hPTP1B (sc-133259) and actin (sc-1616) as well as rabbit anti-goat IgG HRP (sc-2768), goat anti-rabbit IgG HRP (sc-2004) and goat anti-mouse IgG-HRP (sc-2004) antibodies were purchased from Santa Cruz Biotechnology (Dallas, TX). Antibodies against phospho-STAT1 (#7649), STAT1 (#9172), iNOS (#13120), hSTAT1 (#14994), hSTAT1 (#9176) and hPTP1B (#5311) were purchased from Cell Signaling Technology (Danvers, MA). An anti-Flag antibody was purchased from Sigma-Aldrich (Merck KGaA, Darmstadt, Germany).

### Bacterial preparation

*P*. *aeruginosa* strain 8821 was cultured in Luria-Bertani broth at 37°C and harvested when the culture reached an optical density (OD) at 600 nm of 2.5–3 OD units (early stationary phase). Bacteria were washed in phosphate buffer and resuspended in PBS for *in vitro* assays. The *P*. *aeruginosa* strain 8821 (a gift from A. Chakrabarty, University of Illinois, Chicago, IL) used in cell culture assays was killed using an antibiotic mixture (50 U/ml penicillin, 50 U/ml streptomycin, 100 μg/ml piperacillin, 100 μg/ml ceftazidime, and 200 μg/ml gentamycin).

### Phagocytosis assay

Bone marrow-derived neutrophils were isolated from mice following the protocol of the Mouse Neutrophil Negative Selection Kit (STEMCELL Technologies Inc.). *P*. *aeruginosa* 8821 was opsonized with 10% mouse serum for 30 min at 37°C. The neutrophils were counted and then incubated with preopsonized *P*. *aeruginosa* 8821 (multiplicity of infection (MOI) = 10) at 37°C for 30 min. The neutrophil pellet was washed with PBS and then treated with PBS containing 0.1% trypsin and 0.02% EDTA for 15 min at room temperature. Neutrophils were resuspended in PBS containing 10% mouse serum. Specimens were prepared using the Cytospin^™^ 4 Cytocentrifuge (Thermo Fisher Scientific, Waltham, MA). The centrifuged specimens were then stained with a Diff-Quik^™^ staining set (Siemens Healthcare Diagnostics Inc., Newark, DE) and examined under oil immersion. The number of bacteria engulfed by 100 randomly selected neutrophils was counted. The phagocytic activity was measured according to the rate of phagocytosis and the phagocytosis index. The rate of phagocytosis = number of cells containing bacteria/number of cells counted) X 100%. The phagocytosis index = total number of bacteria in all cells/ number of cells counted.

### Intracellular bacterial killing assay

Neutrophils were isolated as described above and incubated with *P*. *aeruginosa* 8821 (opsonized with mouse serum) at 37°C for 1 h. Gentamycin was added at a final concentration of 200 mg/ml for 3 h to kill extracellular bacteria. Then, the neutrophils were washed with PBS and lysed with PBS containing 0.1% Triton X-100. The samples were serially diluted and spread onto Luria broth (LB) agar plates. Colony numbers were determined after an overnight incubation at 37°C.

### Measurement of NO production

Neutrophils were left untreated (NT) or pretreated with TLR4-antagonist (InvivoGen, Catalog#: tlrl-prslps, 10 μg/mL) for 1 h. Then they were left untreated (NT) or exposed to *P*. *aeruginosa* 8821. Cell-free supernatants were collected and analyzed for NO production following the protocol of the Griess Reagent System Kit (Promega, Madison, WI).

### Cytokine production

The concentrations of IL-1β, TNF, IL-6, IFNβ, IP10 and RANTES in culture supernatants were determined by enzyme-linked immunosorbent assay (ELISA) as described previously[24] using DuoSet^®^ Ab pairs from R&D Systems (Minneapolis, MN). Briefly (*e*.*g*. IL-6 ELISA), 96-well plates were coated with an anti-mouse IL-6 antibody for 16–20 h at 4 °C. Nonspecific binding to the plates was blocked using a 1% bovine serum albumin solution in PBS for 1 h at room temperature. A total of 50 μL/well IL-6 standard and samples were added to the plate and incubated for 18–20 h at 4 °C. A biotinylated anti-murine IL-6 antibody was added to each well and incubated for 1 h at room temperature. Streptavidin-HRP (100 μL/well) was added for 30 min at room temperature according to the manufacturer’s instructions. 100 μL/well of 1X TMB Solution was added to each well, and the reaction was stopped with 100 μL Stop Solution (0.5 M H_2_SO_4_). The plate was read at 450 nm and the data was analyzed.

### RNA isolation and qPCR

Total RNA was isolated from neutrophils using TRIzol (Thermo Fisher Scientific) and the RNeasy Mini Kit (Qiagen, Valencia, CA). cDNA was reverse transcribed by using the GoScript^™^ Reverse Transcription System (Promega, Madison, USA). Real-time quantitative PCR was performed with the Bio-Rad CFX-96 Real-Time System. Primer sequences are listed in S1 Table.

### RT^2^ profiling assay

Real-time PCR profiling of mRNAs was conducted with the SYBR Green-based RT^2^ Profiler PCR Array System (Qiagen). Briefly, total RNA was extracted, and first-strand cDNA was synthesized using the RT^2^ First Strand Kit (Qiagen). A PCR primer assay was performed using SYBR Green Supermix (Qiagen) and gene-specific primers that attached to the bottom of the mouse phagocytosis array panel in the CFX96 Real-Time PCR Detection System (Bio-Rad). PCR primer assay data were analyzed on the Qiagen analysis website (www.qiagen.com/us/shop/genes-and-pathways/data-analysis-center-overview-page/), and the scatter plot result was the output.

### Immunoblotting

Cells samples were lysed with RIPA buffer and quantified by using the BCA Protein Assay Kit (Thermo Fisher Scientific). Cell lysates (25 μg) were subjected to electrophoresis on 10% SDS polyacrylamide gels. The proteins were transferred to polyvinylidene difluoride membranes, blotted with primary and secondary antibodies as indicated, and detected by an ECL detection system (SuperSignal^™^ West Pico PLUS Chemiluminescent Substrate, Thermo Fisher Scientific). Scanning densitometry was performed using Scion Image (Scion, Frederick, MD).

### Co-Immunoprecipitation

Co-immunoprecipitation was performed using Protein A/G-agarose beads and then mixed with the relevant antibody. The target proteins were detected using immunoblotting as above.

### Statistics

The data are presented as the means ± SEM of the indicated number of experiments. Statistical significance between multiple treatments was determined by one-way analysis of variance and Tukey’s post hoc honest significance test. Alternatively, when two independent variables were analyzed, two-way analysis of variance and Bonferroni’s multiple-comparison test were used. Statistical analysis was performed using GraphPad Prism software version 5.04 (GraphPad Software Inc., La Jolla, CA). Differences were considered significant at *p < 0.05, **p < 0.01, and ***p <0.001.

## Results

### 1. PTP1B negatively regulates the phagocytosis of *P*. *aeruginosa* by neutrophils

**Fig 1:**
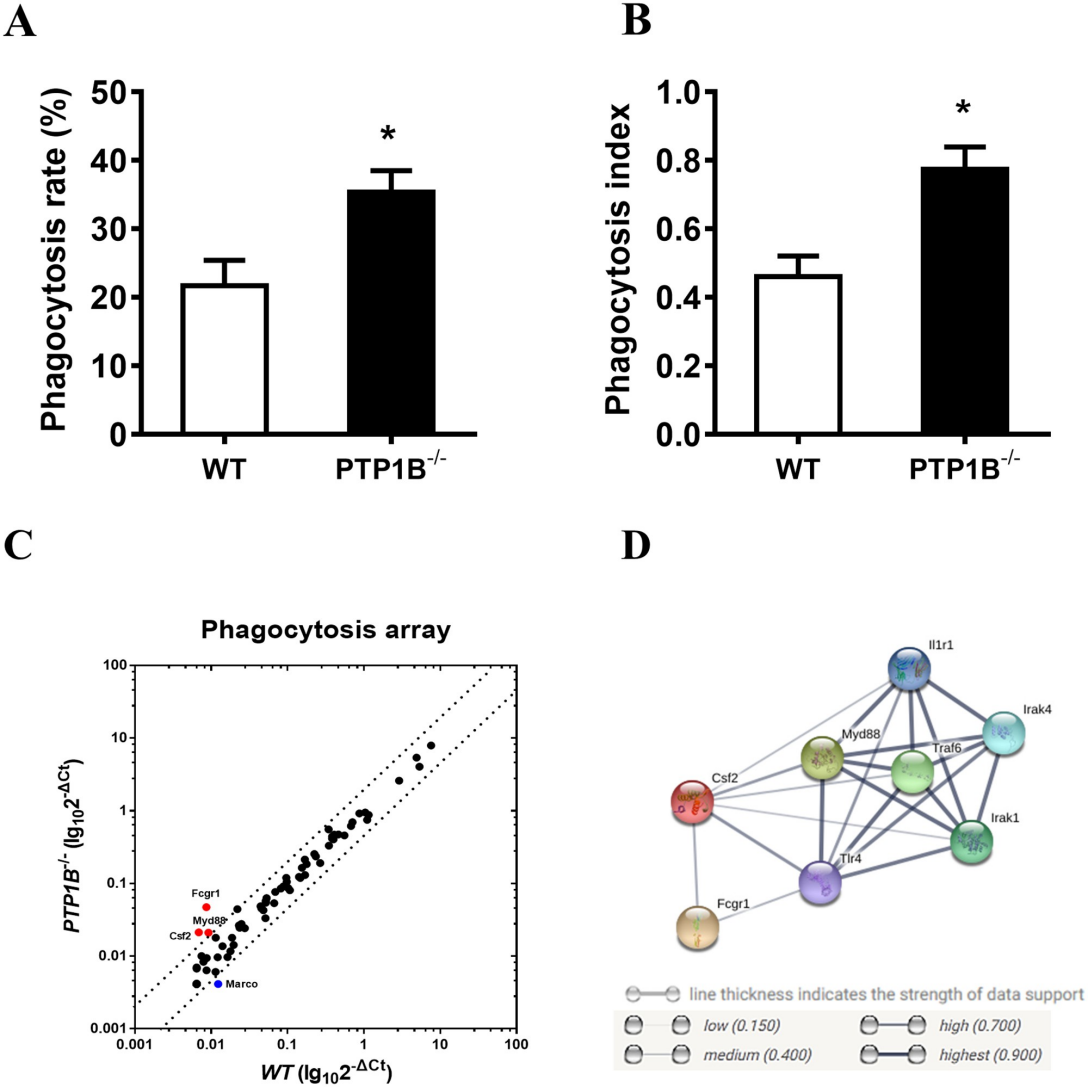
PTP1B deficiency enhances the phagocytosis of *P*. *aeruginosa* by neutrophils. Bone marrow-derived neutrophils were isolated from WT or PTP1B^-/-^ mice, and then incubated with *P*. *aeruginosa* strain 8821 (MOI = 10, opsonized with mouse serum) at 37°C for 30 minutes. Cells were prepared by use of Cytospin^™^, stained with Diff-Quik and examined under a microscope. The number of bacteria engulfed by 100 randomly selected neutrophils was counted. The phagocytic activity was measured according to the rate of phagocytosis (A) and the phagocytosis index (B). (n = 3 ± SEM, *p<0.05) **(C)** Total RNA was isolated. Real-time PCR profiling of mRNAs about phagocytosis were conducted on a RT^2^ Profiler PCR Array System and analyzed on Qiagen analysis website. Red dots indicate increased genes, and blue dots indicate deceased gene (more than twofold change normalized to housekeeping genes). Data are from n = 2 biological replicates. **(D)** Functional prediction of Fcgr1, Myd88, Csf2 were analyzed on STRING website.

To examine whether PTP1B affects the phagocytosis of *P*. *aeruginosa*, bone marrow-derived neutrophils from wild-type or PTP1B-deficient mice were infected with *P*. *aeruginosa* strain 8821. The infected cells were observed under a microscope after Diff-Quik^™^ staining, and the phagocytosis rate (Fig 1A, S1 Fig) and phagocytosis index (Fig 1B) were assessed. The results showed that the phagocytosis ability of PTP1B-deficient neutrophils was significantly increased.

To investigate the effects underlying the negative regulation of PTP1B in phagocytosis, qPCR array assays were performed with the *P*. *aeruginosa*-infected wild-type or PTP1B-deficient neutrophils. The mRNA levels of Fc-gamma receptors (Fcgr1), myeloid differentiation primary response 88 (Myd88) and colony stimulating factor 2 (Csf2) were increased in the PTP1B-deficient neutrophils (Fig 1C). The increased Fcgr1 expression was also confirmed by qPCR (S2A Fig). Then, we used STRING (string-db.org) to predict the functions of the three proteins. In addition to functioning in phagocytosis, Fcgr1, Myd88 and Csf2 may activate TLR4-related signaling (Fig 1D). QPCR also showed that in the PTP1B-deficient neutrophils, the level of TLR4, which is the major innate immune receptor activated by *P*. *aeruginosa*, exhibited a rising trend (S2B Fig). These findings revealed that PTP1B negatively regulates the phagocytosis of *P*. *aeruginosa* by neutrophils.

### 2. Negative regulation of PTP1B on *P*. *aeruginosa* killing by neutrophils

**Fig 2:**
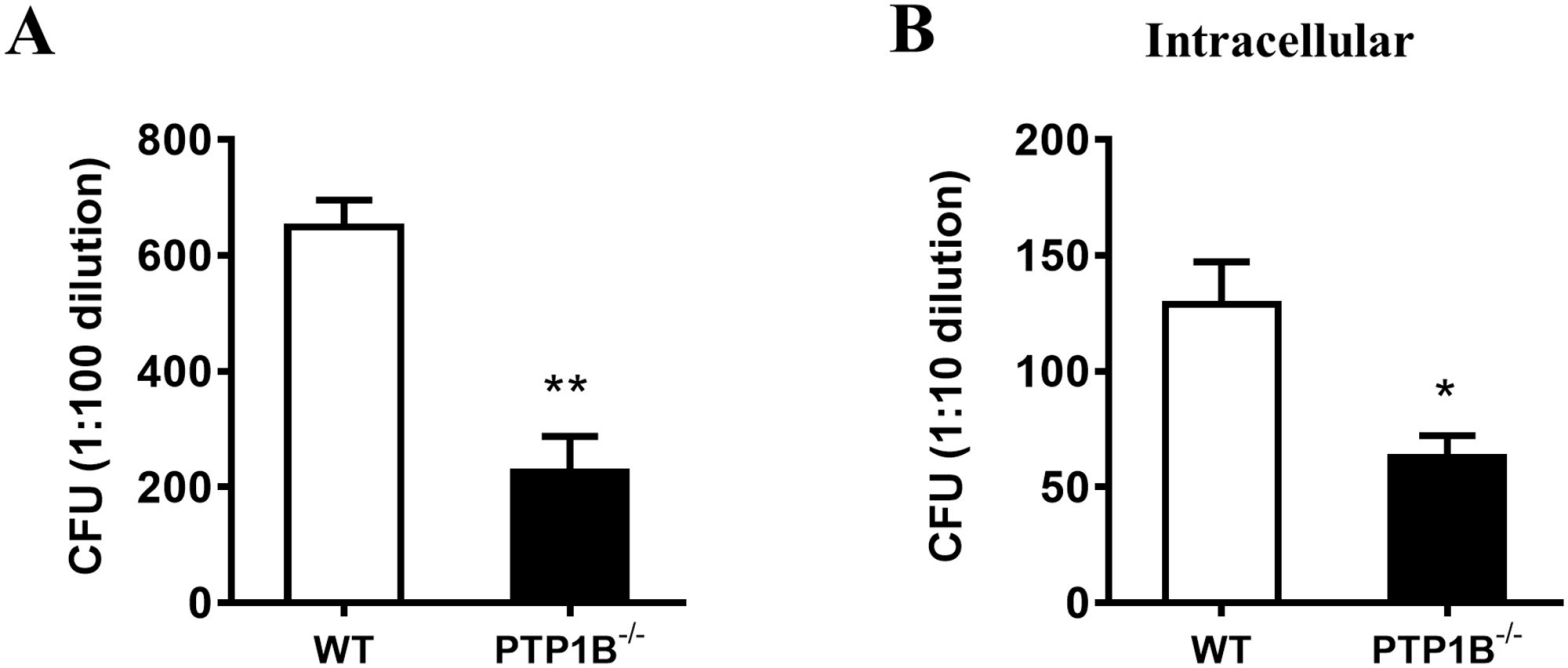
PTP1B deficiency enhances *P*. *aeruginosa*-killing capability of neutrophils. (A) *In vitro* bactericidal assay. Bone marrow-derived neutrophils were isolated from WT or PTP1B^-/-^ mice, and then incubated with *P*. *aeruginosa* strain 8821 (opsonized with mouse serum) at 37°C for 1 hours. Neutrophils were lysed with PBS containing 0.1% Triton X-100. Samples were spread on Luria broth (LB) agar plates. Colony numbers were determined after overnight incubation at 37°C; (B) Intracellular bactericidal assay. In this experiment, gentamicin was added to kill extracellular bacteria. (n = 4 ± SEM, *p<0.05, **p<0.01).

Neutrophils stand at the forefront of innate immunity through their capacities to engulf and kill *P*. *aeruginosa*. Because PTP1B has a negative regulatory effect on phagocytosis, we next examined whether PTP1B affects the killing of *P*. *aeruginosa* by neutrophils. PTP1B-deficient and wild-type neutrophils were infected with *P*. *aeruginosa* strain 8821. Bacterial burden was assessed by CFU counting. Significantly fewer bacteria were detected in the PTP1B-deficient neutrophils compared with that of wild-type neutrophil (Fig 2), suggesting that PTP1B-deficient neutrophils are more efficient in clearing *P*. *aeruginosa*.

### 3. Negative regulation of PTP1B on NO production by neutrophils following *P*. *aeruginosa* infection

**Fig 3:**
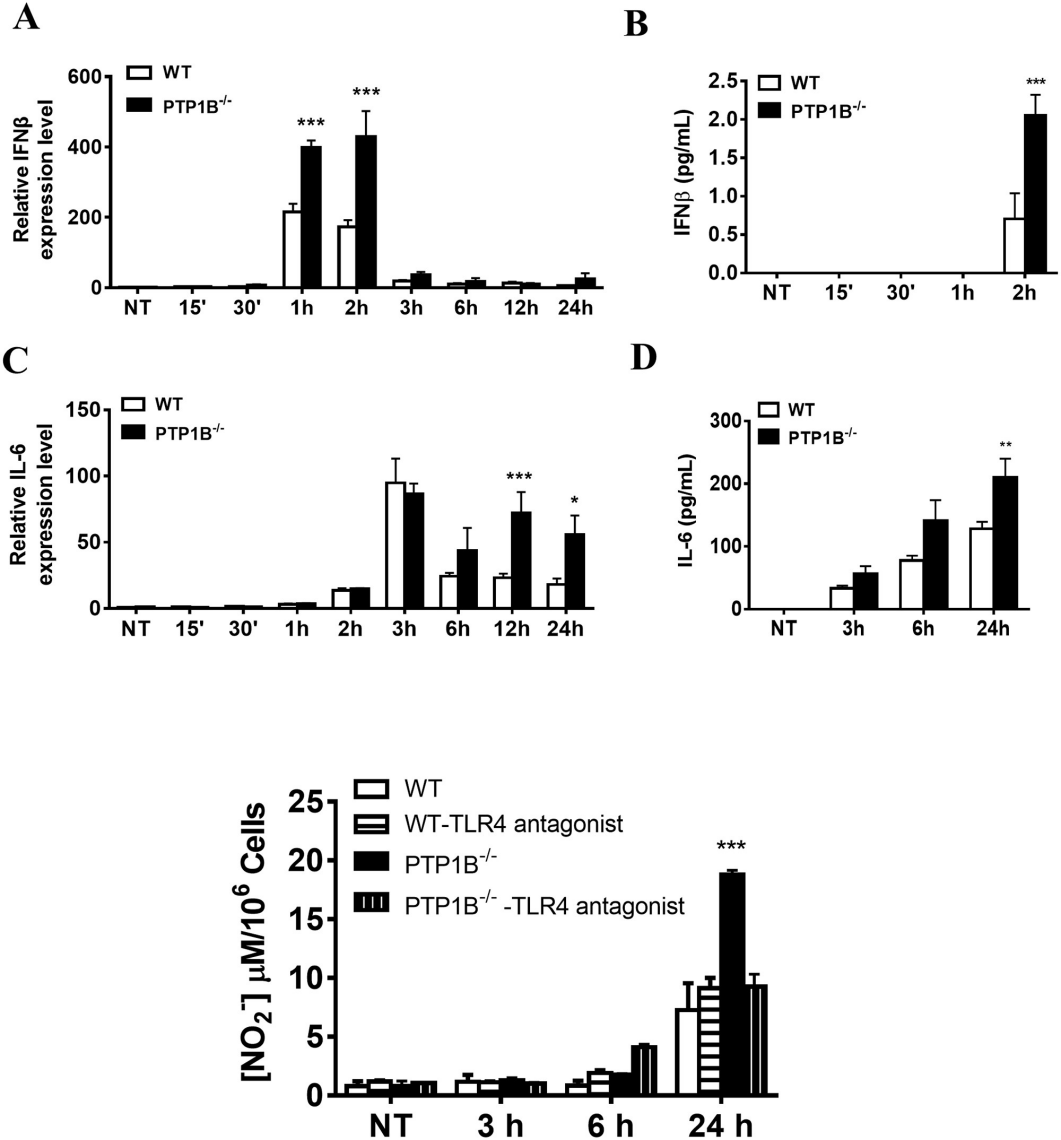
The negative regulation of PTP1B on the production of IFNβ,IL-6 and NO by neutrophil following *P*. *aeruginosa* infection. Wild-type and PTP1B^-/-^ bone marrow-derived neutrophils were left untreated (NT) or exposed to *P*. *aeruginosa* strain 8821 at a MOI10. Total RNA isolated from neutrophils was analyzed by real-time quantitative PCR for IFNβ (A) and IL-6 (C). Supernatants were analyzed by ELISA for the production of IFNβ (B) and IL-6 (D). (n = 3 ± SEM, *p < 0.05, **p < 0.01, ***p < 0.001). (E)Wild-type and PTP1B^-/-^ bone marrow isolated neutrophils were left untreated (NT) or pre-treated with TLR4-antagonist (10μg/mL) for 1h. Then neutrophils were left untreated (NT) or exposed to *P*. aeruginosa strain 8821 at a MOI of 10. Supernatants were analyzed for NO production. (n = 3 ± SEM, ***p < 0.001).

Notably, the cytokines downstream of TLR4-related signaling, such as IL-6 and IFN-β, can induce NO production to facilitate bacterial killing[25]. We found that the expression of both IL-6 and IFN-β increased significantly in the PTP1B-deficient neutrophils (Fig 3A–3D). PTP1B deficiency has no effect on the production of IP10, RANTES, TNF and IL-1β by neutrophil following *P*. *aeruginosa* infection (S3 Fig). Subsequently, we analyzed NO production in the PTP1B-deficient neutrophils upon *P*. *aeruginosa* challenge and showed that it was negatively regulated by PTP1B in a TLR4-dependent manner (Fig 3E).

### 4. PTP1B activates TLR4-STAT1-iNOS signaling

**Fig 4:**
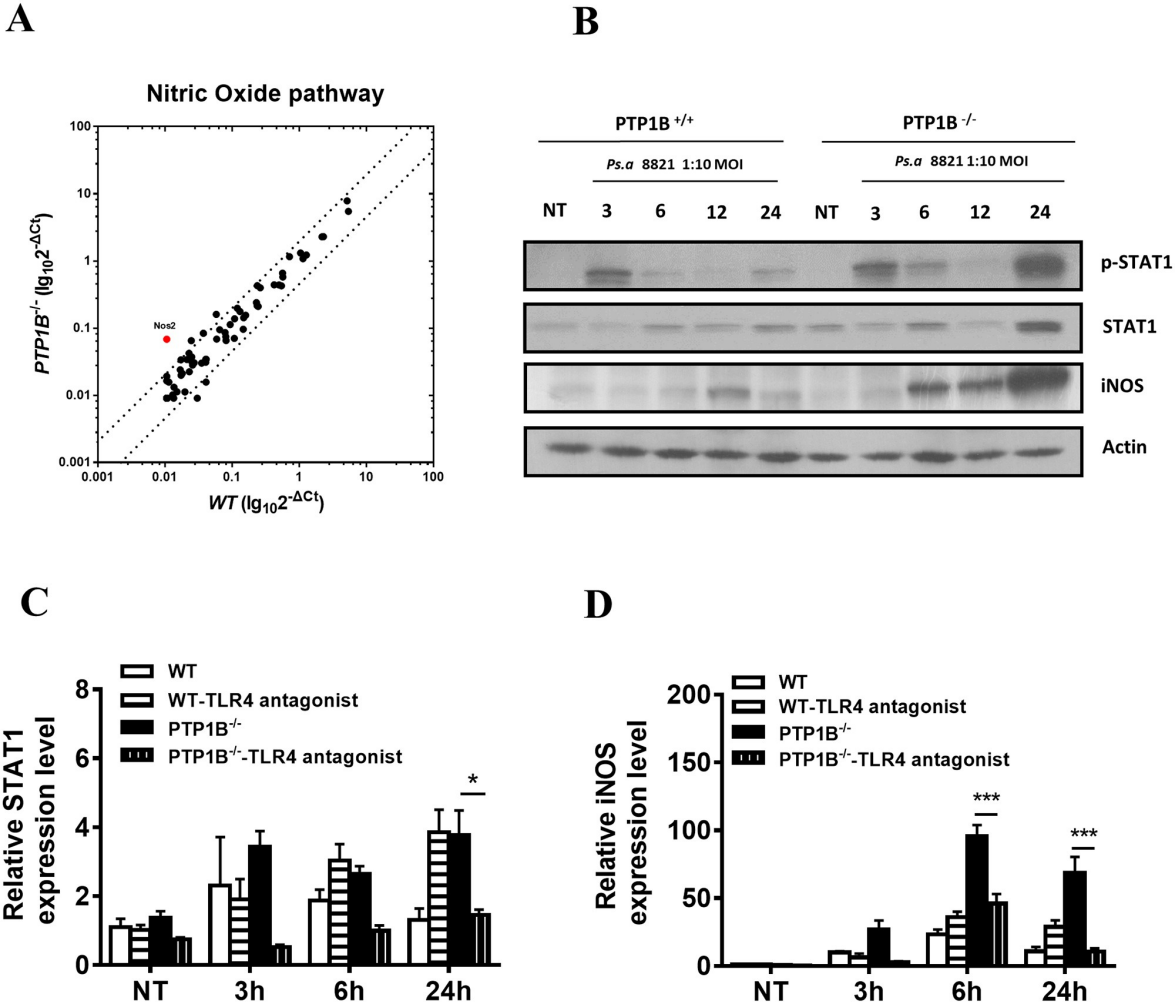
PTP1B regulates TLR4-STAT1-iNOS pathway activation. Wild-type and PTP1B^-/-^ bone marrow-derived neutrophils were left untreated (NT) or exposed to *P*. *aeruginosa* strain 8821 at a MOI of 10 for 3, 6, 12 and 24 h. (A) Total RNA of 3 h was isolated. Real-time PCR profiling of mRNAs about Nitric Oxide pathway were conducted on a RT^2^ Profiler PCR Array System and analyzed on Qiagen analysis website. Red dots indicate increased genes (more than twofold change normalized to housekeeping genes). Data are from n = 2 biological replicates. (B) Lysates were prepared and subjected to Western blot for p-STAT1, STAT1, iNOS and actin. Blots are representative of three separate experiments. (C, D) Wild-type and PTP1B^-/-^ bone marrow-derived neutrophils were pre-treated with TLR4-antagonist (10 μg/mL) for 1h. Then neutrophils were left untreated (NT) or exposed to *P*. *aeruginosa* strain 8821 at a MOI of 10 for 3h, 6h and 24 h. Total RNA were analyzed by real-time quantitative PCR for STAT1 (C) and iNOS (D). STAT1 and iNOS expression was normalized by using actin as an endogenous control. The average value of iNOS and STAT1 at the NT-WT (no *P*. *aeruginosa* infection in wild-type neutrophil) was used as a calibrator to determine the relative levels of iNOS and STAT1 at different conditions. Data are the mean of 4 mice per group. (n = 4 ± SEM, **p < 0.01).

Cytokine-activated NO production involves a variety of components. It is unclear whether these molecules are regulated by PTP1B. To address this question, qPCR arrays were used to assess the NO pathway. The results revealed that only the iNOS (NOS2) mRNA levels significantly increased (Fig 4A), which was corroborated by protein abundance analysis using Western blotting (Fig 4B). INOS, whose expression is modulated by the upstream transcription factor STAT1, induces NO production[26]. The data revealed that STAT1 phosphorylation and expression levels were both negatively regulated by PTP1B (Fig 4B). And the PTP1B regulated expression of STAT1 and iNOS could be blocked by TLR4 antagonist (Fig 4C and 4D). Our findings indicated that the TLR4-STAT1-iNOS axis is the main signaling pathway negatively regulated by PTP1B in NO-mediated bacterial killing.

### 5. STAT1 is the target of PTP1B

**Fig 5:**
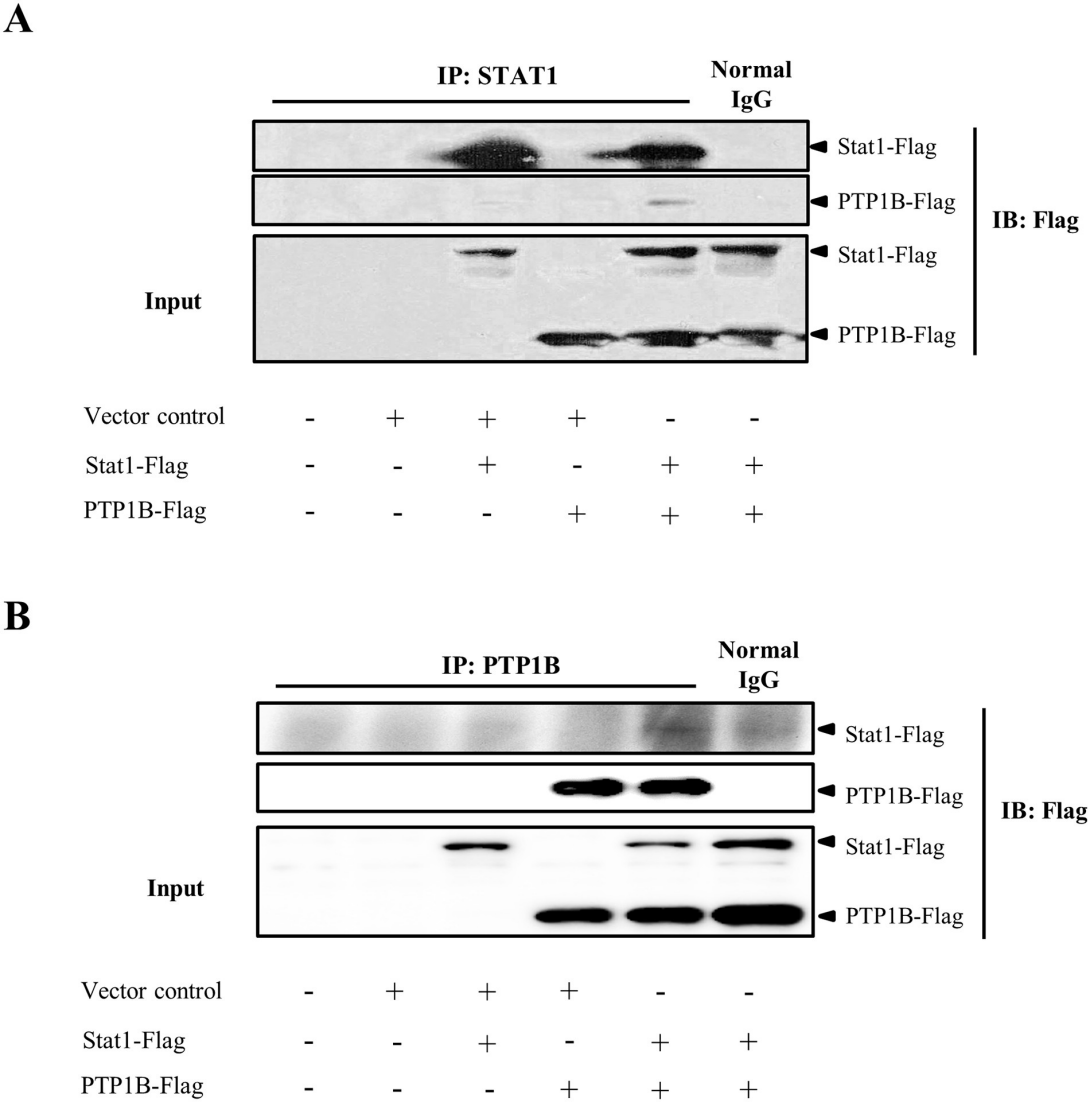
STAT1 is the target of PTP1B. HEK293 cells were transfected with plasmids encoding STAT1, PTP1B. Cell lysates were immunoprecipitated for STAT1 (A) or PTP1B (B) and blotted for the Flag-tag. Blots are representative for two independent experiments.

STAT1 is activated by tyrosine phosphorylation, and whether it is the target of PTP1B has not been reported previously. In this study, the interaction between PTP1B and STAT1 was verified by an immunoprecipitation assay. Specifically, PTP1B and STAT1 were expressed in HEK293 cells. Pulling down either PTP1B or STAT1 demonstrated that the two proteins interacted with each other (Fig 5A and 5B), which was relieved after *P*. *aeruginosa* infection (S4 Fig). Together, these results demonstrated that the targeting of STAT1 by PTP1B facilitates the PTP1B-mediated negative regulation of the neutrophil killing of *P*. *aeruginosa* by the TLR4-STAT1-iNOS pathway.

## Discussion

*P*. *aeruginosa* causes pulmonary infection in immune-compromised individuals and leads to tissue damage or a decline in lung function[27]. Hence, a calculated innate immune response is essential for eliminating *P*. *aeruginosa* infection. Protein tyrosine phosphatase plays an important role in the host immune response against pathogen infection by regulating phosphorylation[28]. We previously reported that PTP1B negatively regulates *P*. *aeruginosa* lung infection through the interferon I pathway[23]. Neutrophil infiltration in lungs of PTP1B-deficient mice significantly increased, which may contribute to effective pulmonary bacterial clearance[23]. However, the mechanism remained incompletely understood. This study revealed that PTP1B targets STAT1 and regulates the NO-mediated clearance of *P*. *aeruginosa* by neutrophils. Our results not only showed that PTP1B is responsible for the negative regulation of the TLR4-STAT1-iNOS signaling pathway but also demonstrated the interaction between PTP1B and STAT1.

Neutrophils are the first line of defense against bacterial infection. During the mobilization of the immune system, neutrophils take the lead to reach the inflammatory site of *P*. *aeruginosa* infection and kill the bacteria. As such, they constitute a pivotal component of the acute inflammatory response[29]. Neutrophils express a variety of receptors. By opsonization, Fcgr1 mediates the phagocytosis of bacterial-antibody complexes by neutrophils. Our study revealed that phagocytosis was enhanced in PTP1B-deficinet neutrophils and the expression of Fcgr1 was increased. In addition, *P*. *aeruginosa* activated the innate immune signaling pathway through TLRs and increased the expression level of bactericidal components. This study focused on the RNS pathway. NO, as an important effector of the RNS pathway, is one of the most important compounds produced by neutrophils for eliminating bacteria. In the immune system, the activation of neutrophils results in the generation of iNOS, which catalyzes L-arginine to produce NO. In addition, the TLR signaling pathway activates iNOS production through multiple transcription factors[30]. The regulatory mechanisms of this process remain unclear. We provide strong evidence showing that PTP1B is a key regulator.

We previously reported that macrophages[31], mast cells[32] and dendritic cells[23] play crucial roles in *P*. *aeruginosa* infection. Recently, our study revealed that platelet-binding neutrophils were involved in the clearance of *P*. *aeruginosa* in the lungs[24]. These studies suggest that different immune cells play distinct roles in host defense against *P*. *aeruginosa* infection. We have demonstrated that the regulation of PTP1B in *P*. *aeruginosa*-infected mice is mainly concentrated on the TRIF-IRF-IFN signaling pathway and that a similar trend is also observed in dendritic cells *in vitro*[23]. In this study, we found that IL-6 and IFN-β were negatively regulated by PTP1B. These results not only indicated that PTP1B can selectively modulate the expression of some downstream cytokines in neutrophils, but also suggested that the downstream signals activating these two cytokines may contribute to the neutrophil-mediated elimination of *P*. *aeruginosa*.

JAK/STAT signaling is usually stimulated by cytokines, such as IL-6, IFN-β, and TNF. It is widely involved in various immunopathological processes, including cancer development and pathogen infection[33]. STAT1[34], STAT3[35] and STAT6[36] are important components of immune responses and inflammation. We previous demonstrated the unique role of STAT4 in innate immunity in *P*. *aeruginosa* infection[37]. PTP1B is an important molecule in modulating the JAK/STAT signaling pathway. In PTP1B-deficient mice, the dysregulation of the JAK/STAT signaling pathway is the main cause of some immune dysfunction[38]. STAT3[39] and STAT6[40] are the substrates of PTP1B. STAT1 is a major connection between the two canonical TLRs and the JAK/STAT pathways[41], but its role in antibacterial immunity has not been well understood. In addition, although STAT1 can also be activated by tyrosine phosphorylation, whether it is the target of PTP1B has not been reported. Herein, we report for the first time that STAT1, as the target of PTP1B, is involved in bacterial clearance. This finding reminds the important roles of STATs in counteracting pathogen infection[42].

Considering the role of PTP1B in the neutrophil-mediated elimination of *P*. *aeruginosa*, PTP1B inhibitors have therapeutic potential[43]. Although the properties of PTP1B have made it difficult to investigate this protein, in recent years a number of breakthrough achievements have been accomplished in the research and development of PTP1B inhibitors[44]. Meanwhile, we will monitor the efficacy of these inhibitors in clinical application.

## Supporting information

S1 Fig PTP1B deficiency enhances the phagocytosis of *P*. *aeruginosa* by neutrophils and has no effect on cell number and purity. Bone marrow-derived neutrophils were isolated from WT or PTP1B^-/-^ mice. Cells were counted by haemocytometer (A), and then incubated with *P*. *aeruginosa* strain 8821 (MOI = 10, opsonized with mouse serum) at 37°C for 30 minutes. Cells were prepared by use of Cytospin^™^, stained with Diff-Quik and examined under a microscope (B, C).
(TIF)

S2 Fig PTP1B-deficient neutrophils display activated *Fcgr1* and *tlr4* transcription following *P*. *aeruginosa* infection. Wild-type and PTP1B^-/-^ bone marrow-derived neutrophils were left untreated (NT) or exposed to *P*. *aeruginosa* strain 8821 (MOI = 10) for 3h, 6h, 12h and 24 h. Total RNA isolated neutrophils were analyzed by real-time quantitative PCR for *fcgr1* (A) and *tlr4* (B). The expression was normalized by using *hprt* as an endogenous control. The average value of *fcgr1* and *tlr4* at the NT-WT (no *P*. *aeruginosa* infection in wild-type neutrophil) was used as a calibrator to determine the relative levels of *fcgr1* and *tlr4* at different conditions. Data are the mean of 4 mice per group. (n = 4 ± SEM, **p < 0.01, ****p < 0.0001).
(TIF)

S3 Fig PTP1B deficiency has no effect on the production of IP10, RANTES, TNF, and IL-1β by neutrophil following *P*. *aeruginosa* infection. Wild-type and PTP1B^-/-^ bone marrow-derived neutrophils were left untreated (NT) or exposed to *P*. *aeruginosa* strain 8821 (MOI = 10) for 15’, 30’, 1h, 2h, 3h, 6h, 12h and 24 h. Total RNA isolated was analyzed by real-time quantitative PCR for IP10 (A), RANTES (B), TNF (C) and IL-1β (D). (n = 3 ± SEM).
(TIF)

S4 Fig The interaction of STAT1 and PTP1B is relieved after *P*. *aeruginosa* infection. HEK293 cells were transfected with plasmids encoding STAT1 or PTP1B following *P*. *aeruginosa* strain 8821 infection for 4 hours (MOI = 10). Cell lysates were immunoprecipitated for STAT1 (A) or PTP1B (B) and blotted for the Flag-tag. Blots are representative for two independent experiments.
(TIF)

S1 Table Primers for qPCR. (DOCX)
