# Supplementary figures and images for: PTP1B negatively regulates nitric oxide-mediated Pseudomonas aeruginosa killing by neutrophils
Source: PLoS One. 2019 Sep 18;14(9):e0222753. doi: 10.1371/journal.pone.0222753 (PMC6750887; doi:10.1371/journal.pone.0222753)

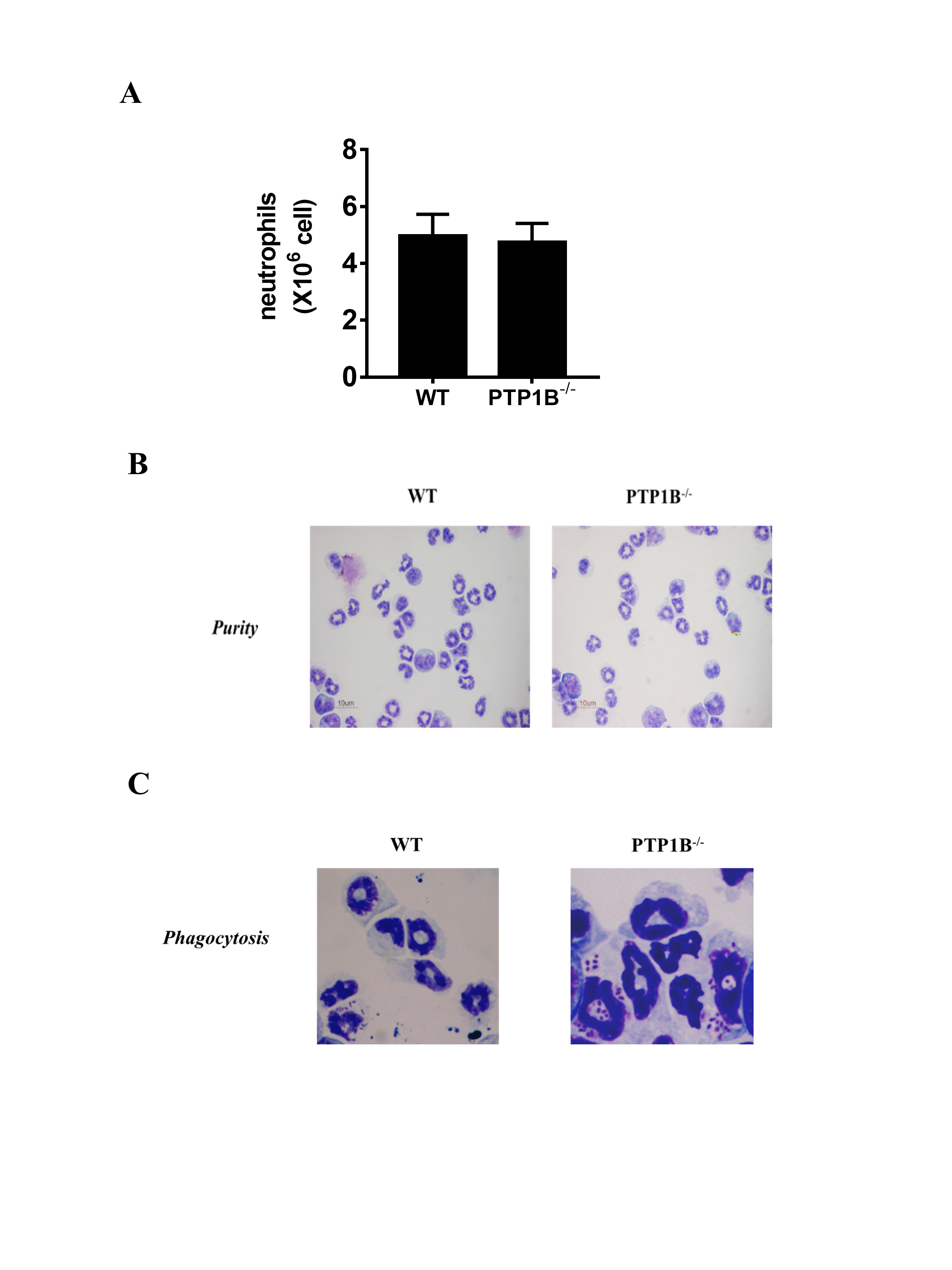

Supplement: S1 Fig — Bone marrow-derived neutrophils were isolated from WT or PTP1B-/- mice. Cells were counted by haemocytometer (A), and then incubated with P. aeruginosa strain 8821 (MOI = 10, opsonized with mouse serum) at 37°C for 30 minutes. Cells were prepared by use of Cytospin™, stained with Diff-Quik and examined under a microscope (B, C). (TIF) [file pone.0222753.s001.TIF]

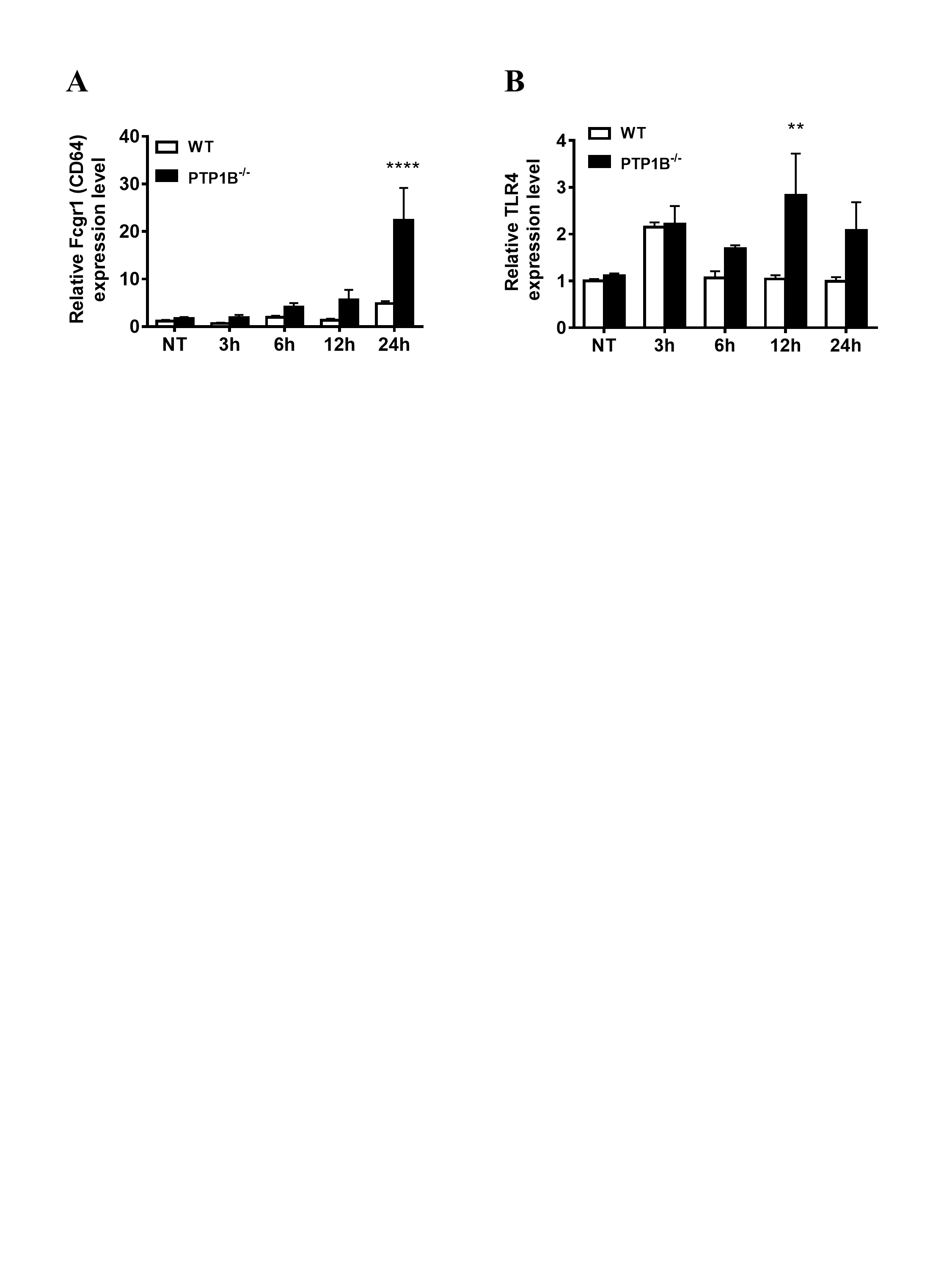

Supplement: S2 Fig — Wild-type and PTP1B-/- bone marrow-derived neutrophils were left untreated (NT) or exposed to P. aeruginosa strain 8821 (MOI = 10) for 3h, 6h, 12h and 24 h. Total RNA isolated neutrophils were analyzed by real-time quantitative PCR for fcgr1 (A) and tlr4 (B). The expression was normalized by using hprt as an endogenous control. The average value of fcgr1 and tlr4 at the NT-WT (no P. aeruginosa infection in wild-type neutrophil) was used as a calibrator to determine the relative levels of fcgr1 and tlr4 at different conditions. Data are the mean of 4 mice per group. (n = 4 ± SEM, **p < 0.01, ****p < 0.0001). (TIF) [file pone.0222753.s002.TIF]

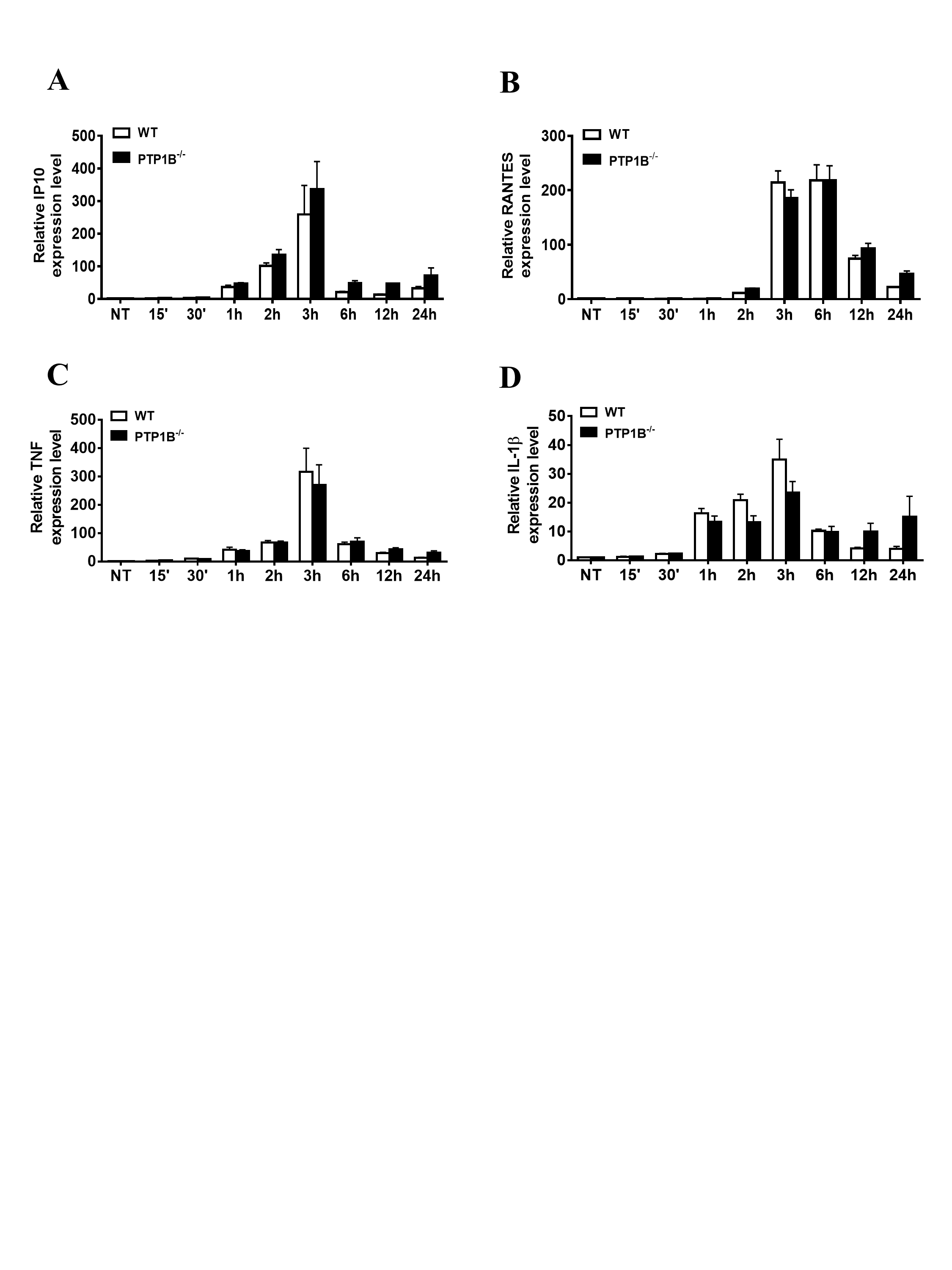

Supplement: S3 Fig — Wild-type and PTP1B-/- bone marrow-derived neutrophils were left untreated (NT) or exposed to P. aeruginosa strain 8821 (MOI = 10) for 15’, 30’, 1h, 2h, 3h, 6h, 12h and 24 h. Total RNA isolated was analyzed by real-time quantitative PCR for IP10 (A), RANTES (B), TNF (C) and IL-1β (D). (n = 3 ± SEM). (TIF) [file pone.0222753.s003.TIF]

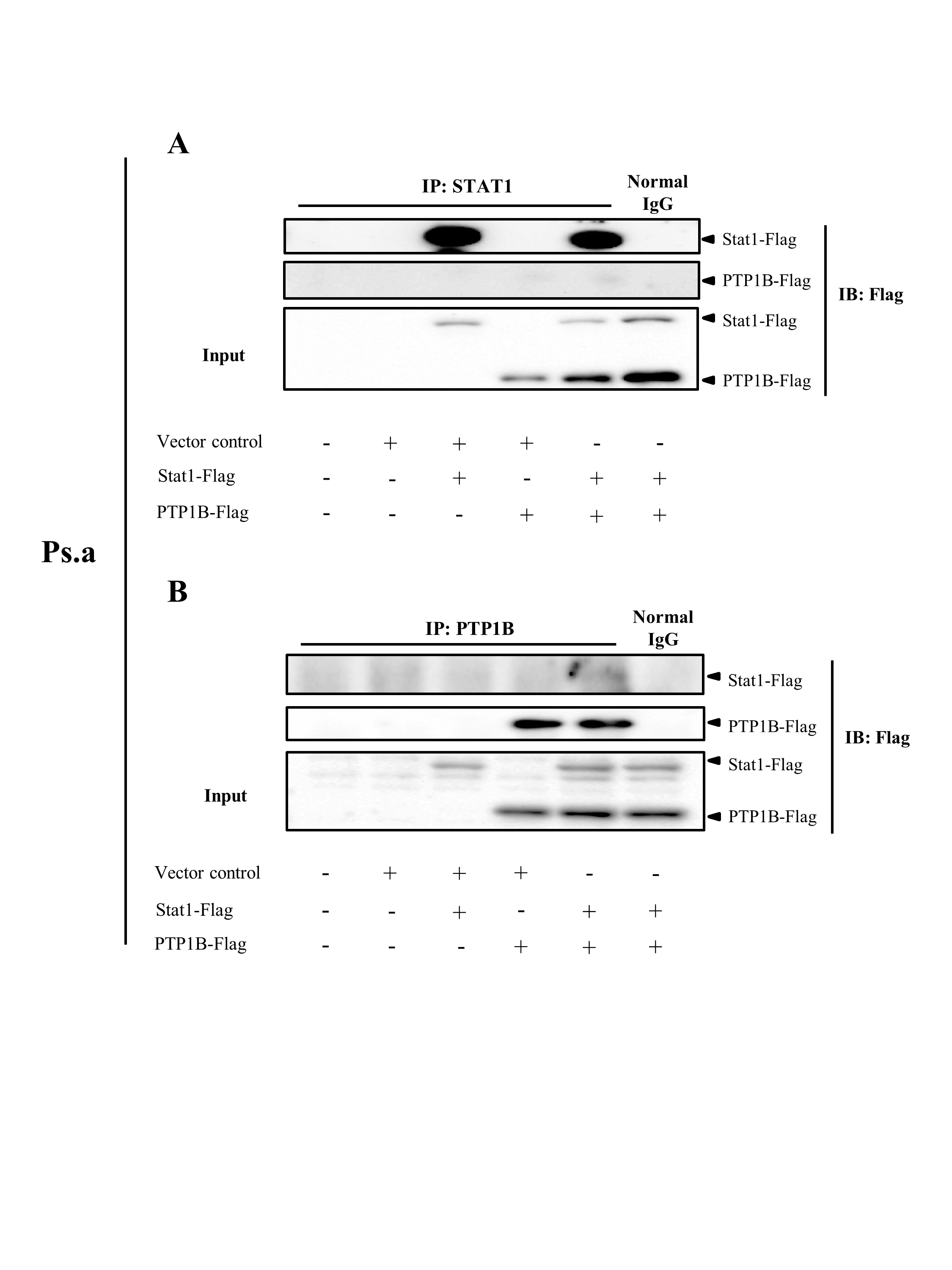

Supplement: S4 Fig — HEK293 cells were transfected with plasmids encoding STAT1 or PTP1B following P. aeruginosa strain 8821 infection for 4 hours (MOI = 10). Cell lysates were immunoprecipitated for STAT1 (A) or PTP1B (B) and blotted for the Flag-tag. Blots are representative for two independent experiments. (TIF) [file pone.0222753.s004.TIF]
