# Supplementary material for: PTP1B negatively regulates nitric oxide-mediated Pseudomonas aeruginosa killing by neutrophils
Source: PLoS One. 2019 Sep 18;14(9):e0222753. doi: 10.1371/journal.pone.0222753 (PMC6750887; doi:10.1371/journal.pone.0222753)
Supplement: S1 Table — (DOCX) [file pone.0222753.s005.docx]

Supplementary Table

Table S1: Primers for qPCR

| **Genes** | **Forward Primers** | **Reverse Primers** |
| --- | --- | --- |
| iNOS | GAGACAGGGAAGTCTGAAGCAC | CCAGCAGTAGTTGCTCCTCTTC |
| STAT1 | GCCTCTCATTGTCACCGAAGAAC | TGGCTGACGTTGGAGATCACCA |
| TLR4 | AGCTTCTCCAATTTTTCAGAACTTC | TGAGAGGTGGTGTAAGCCATGC |
| CD64 | ACCTGAGTCACAGCGGCATCTA | TGACACGGATGCTCTCAGCACT |
| IL-6 | TGGTACTCCAGAAGACCAGAGG | AACGATGATGCACTTGCAGA |
| IFNβ | GCCTTTGCCATCCAAGAGATGC | ACACTGTCTGCTGGTGGAGTTC |
| TNFα | ATGAGAGGGAGGCCATTTG | CAGCCTCTTCTCATTCCTGC |
| IL-1β | AGGTCAAAGGTTTGGAAGCA | TGAAGCAGCTATGGCAACTG |
| IP10 | ATCATCCCTGCGAGCCTATCCT | GACCTTTTTTGGCTAAACGCTTTC |
| RANTES | TACACCAGTGGCAAGTGCTC | ACACACTTGGCGGTTCTTTC |
| HPRT | CTCATGGACTGATTATGGACAGGAC | GCAGGTCAGCAAAGAACTTATAGCC |
